# Bibliographical

**Published:** 1886-10

**Authors:** 


### BIBLIOGRAPHICAL.

THE AMERICAN SYSTEM OF DENTISTRY. In treatises by various authors. Edited by WILBUR F. LITCH, M. D., D.D. S., Professor of Prosthetic Dentistry, Therapeutics and Materia Medica, in the Pennsylvania College of Dental Surgery.

Volume I. Regional and Comparative Dental Anatomy; Dental Histology and Dental Pathology. Philadelphia: Lea Brothers & Co. 1886.

This is the first volume of a work that has been in the course of preparation for some time, and concerning which considerable curiosity has been manifested. It consists of a series of papers, the most of them original, but some of which are reprints from dental journals and other sources. The plan pursued is to submit each subject to some one who, in the opinion of the editor, is especially well qualified to speak concerning it, and then to present the papers in the author's own words. This has, in the first volume, given us a series of excellent essays, which contain the results of the latest observations of each writer in the several fields covered by the volume. Whether the final issue will be a complete "system" of dentistry, with a unity of purpose and a harmony in execution that shall entitle it to rank as a standard text-book, is a question which can only be decided when the last volume is before us. It would be manifestly improper to pass judgment upon it as a whole, until it can be examined in its completed state. It is certain, however, that every progressive dentist will wish to add it to his library, for purposes of examination and reference, if for nothing more.

The contents of Vol. I, are as follows :

Regional Anatomy, etc. By M. H. Cryer, M. D., D.D.S.

Lymphatic Vessels of the Head and Neck. By Albert P. Brubaker, A. M., M. D., D.D.S.

The Teeth of the Invertebrates. By W. H. Dall.

The Teeth of the Vertebrates. By Jacob L. Wortman, M. D.

Embryology and Histology. By W. Xavier Sudduth, M. D., D.D.S.

General Pathology. By G. V. Black, M. D., D.D.S.

Dental Caries. Same author.

Pathology of the Dental Pulp. Same author.

Diseases of the Peridental Membrane. Same author.

Abrasion and Erosion of the Teeth. Same author.

Diseases of the Dental Pulps and their Treatment. James Truman, D.D.S.

Dr. W. D. Miller's series of papers, first published in the INDEPENDENT PRACTITIONER, "Fermentation in the Human Mouth," and "Biological Studies of Fungi in the Human Mouth," are republished entire, with the original illustrations given in this journal.

The first volume is a very handsome one of 1,034 octavo pages, profusely illustrated, many of the cuts, however, being reproduced from other authors.

DICTIONARY OF PRACTICAL SURGERY. By various British Hospital Surgeons. Edited by CHRISTOPHER HEATH, F. R. C. S. Philadelphia: J. B. Lippincott Co. 1886.

The name of Prof. Heath is familiar to surgeons in this country—more especially to dental surgeons—from his well-known work on "Injuries and Diseases of the Jaws," the first edition of which was published in 1868, and which has since remained the leading text-book in its special field. The present work is of a more ambitious character, covering the whole of surgery. It is intended as a book of reference for the busy practitioner, who is constantly meeting emergencies in which he desires immediate information as to diagnosis and treatment, and in which there is not time for a study of extended treatises.

The surgical affections are arranged in an alphabetical form, and the subjects are considered as to Cause, Pathology, Symptoms, and Diagnosis, Treatment and Prognosis. Almost every surgical disease or injury that can be conceived of is thoroughly considered, and there are references for allied affections, which enable the examiner readily to compare them with each other. There is also a copious index, which enables the student easily to find any particular article which he may desire to consult.

The book will be indispensable to every dental surgeon, for there are many excellent articles which pertain to his specialty. Prof. Heath himself contributes a number on Diseases of the Jaws, of the Gums, Operations in the Oral Cavity, Dentigerous Cysts, Fractures of the Alveolus, Diseases of the Antrum, etc., etc.

Oakley Coles writes on Mechanical, and Thomas Smith on Surgical treatment of Cleft Palate. J. W. Haward furnishes articles on Parotid Tumors, Salivary Fistulas, etc. W. M. Baker on Diseases of the Tongue, etc. W. W. Cheyne on Inflammation and Antiseptics, etc. Stephen Paget on Cancrum Oris, etc. A. J. Pepper on Diseases of the Lips, and Charles Tomes on Caries of the Teeth, Dentition, Extraction, Teeth as a Test of Age, Toothache, etc. Besides these there are hundreds of other articles of equal value to the surgeon. It will be readily comprehended, then, that this Dictionary of Surgery will fill an important place in the library of every dentist who desires thoroughly to qualify himself for the practice of his whole profession.

It is a handsome octavo volume of nearly 900 pages, and we most unhesitatingly commend it to every dentist as one of the most useful books of reference with which he can provide himself.

Medical and Surgical Directory of the United States. R. L. Polk & Co., 280 Broadway, New York City. 1886.

We cannot give a better idea of the design and scope of this most excellent and useful compendium, than by quoting the list of contents from the title page. It comprises "Lists of Physicians and Surgeons, arranged by States, with School practiced, Post-office address, population and location, date and College of graduation; all the existing and extinct Medical Colleges in the United States and Canada, with Location, Officers, Number of Professors, Lecturers, Demonstrators, etc.; the various Medical Societies, Medical Colleges, Hospitals, Sanitariums, Asylums, and other Medical Institutions, Boards of Health; a Synopsis of the Laws of Registration, and other Laws relating to the Profession in each State; Medical Journals, with names of editors, frequency of publication and subscription rates; Official List of Officers of the Medical Department of the U. S. Army, Navy, and Marine Hospital Service; Roster of Examining Surgeons of the U. S. Pension Department; a descriptive sketch of each State and Territory, embodying such matters as location, boundaries, extent in miles and acres, latitude and longitude; statistics relating to Climate, Temperature, rate of Mortality, number of deaths from consumption, etc.; the names and location of all the best known Mineral Springs; full particulars of all National Associations and Societies relating to Medicine and Surgery, and a list of all Physicians in the United States, arranged alphabetically, with the number of the page on which the name occurs."

If any one can think of any other information which such a book should contain, it will, without doubt, be found. The whole forms a handsome octavo volume of 1,400 pages, and is a complete encyclopedia of medical statistics and information.

DENTAL SCIENCE. QUESTIONS AND ANSWERS. A compendium of Lectures on Dental Materia Medica, Dental Physiology, Dental Pathology and Therapeutics. By Luman C. Ingersoll, A. M., D. D. S., Dean of the Dental Department of the University of Iowa. L. C. INGERSOLL, Publisher, Keokuk, Iowa.

This excellent work is the result of thirty years of experience at the operating chair, and of long service as a writer and teacher, by one of the most thoughtful, able, and studious dentists of America. There is not a dental society in our land, of any prominence, which does not know Dr. Ingersoll by his writings or speeches, and if there be an intelligent dentist in the profession who has not been assisted by his wise counsels and instructions, it is because he is not acquainted with dental literature. The book contains that which Dr. Ingersoll has spent a life-time in acquiring. It is the result of original investigations, and its every page evidences that its author is a thinking man, It is more comprehensive than the most of works of the kind, and contains a mass of information that is not usually incorporated in them. Its price is but $2.00, and in buying it the dental reader may rest assured that he will view the truth from another standpoint than that which is taken by the conventional writer. We advise every student to get it by all means, and then carefully to study it.

EPITOME OF DISEASES OF THE SKIN. By LOUIS A. DUHRING, M. D. Philadelphia: J. B. Lippincott Co. 1886.

This little volume is an abstract of a course of lectures delivered in the University of Pennsylvania, during the sessions of '83 and

'84. They were admirably reported by Henry Wile, M. D., in abstract, for the *Medical News*, and are now revised and published in book form, and make a very useful and convenient handbook of the subject.

Transactions of the New York Odontological Society for 1885. Philadelphia: The S. S. White Dental Manufacturing Co. 1886.

We have before spoken in commendation of the volumes of the proceedings of this excellent society. The present forms another quite as handsome and of equal interest with its predecessors.

Transactions of the Illinois State Dental Society for 1886. Chicago: H. D. Justi.

Of all the many dental societies in the United States, there are few that obtain from their own members so much of strictly original matter as does the Illinois State. The present volume comprises some of the best papers that have issued from the same source, and while there are many things contained within it to which not every intelligent man will give his assent, there is nothing the perusal of which will not do him good.

Report of the Second Annual Meeting, Alumni Association of the Chicago College of Dental Surgery.

There were many capital hits in some of the addresses made upon this occasion, and if we had the space we would like to make some extracts from the responses of Dr. E. E. Cady and Dr. J. D. Moody.

The Neurological Review. Edited by J. S. Jewell, M. D. Monthly. Rand, McNally & Co., publishers, Chicago.

This is a new journal devoted to Neurological Science, which has already taken a high position in professional literature, and gives promise of great usefulness. We most cordially welcome it to our exchange list, and hope that its success will be commensurate with its merits, in which case its publishers will have little cause for regrets.

Diet Tables. New York: Reed & Carnrick.

This is a capital little hand and reference book, giving in most convenient form the dietary most appropriate in some of the more common diseases, like diabetes, dysentery, phthisis, etc. It also tells what should be avoided. Any one can readily see the great advantages in the possession of such a work. The leaves are perforated for tearing out and marking for the guidance of each patient.

*The Treatment and Filling of Root Canals at a Single Sitting.* By C. T. STOCKWELL, D. D. S., Springfield, Mass.; also *A Method of Saving Badly Broken Soft Teeth.* By the same author.

Dr. Stockwell is a vigorous writer, and in these papers read before the Vermont State Dental Society, he is at his best. While we cannot agree with him in all his statements or deductions, we have read the papers with much pleasure and profit. We like an author who gives some opportunity for debate, and who does not follow the beaten track of conventional treatment.

*Some Recent Experiences in Clinical Surgery.* By DONALD MACLEAN, M. D. Reprint from the Transactions of the Mich. State Medical Society.

*Esthetics of Medicine.* By H. A. COTTELL, M. D. Reprint from the *American Practitioner and News.*

*Electrolysis in Gynecology.* With reports of cases and discussions. By FRANKLIN H. MARTIN, M. D. Reprint from the *Jour. Am. Med. Association.*

*Address in Dental and Oral Surgery.* By JOHN S. MARSHALL, M. D., Chicago. Reprint from the *Jour. Am. Med. Association.*

*The Progressive and Retrogressive Physiological Metamorphosis of the Jaws and Teeth.* By DR. JOHN J. R. PATRICK, Belleville, Ill. Reprint from the *Dental Cosmos.*

*Dental Spiritualism.* By DR. LOUIS OTTOFY, Chicago, Ill. Reprint from the Illinois State Dental Society Transactions.

*Intubation of the Larynx for Diphtheritic Croup.* By E. FLETCHER INGALLS, A. M., M. D. Reprint from the *Jour. Am. Med. Association.*

*First Annual Report of the Board of Dental Examiners of Kansas.*

*The Limitation of the Gangrenous Stage of Syphilis.* By F. N. Otis, M. D. Reprint from *Jour. Cutaneous and Venereal Diseases.*

*Report of Committee on Dental Science and Literature.* By Dr. C. R. E. Koch, Chicago. Reprint from the Transactions of the Illinois State Dental Society.

*The Legal Status of Medical Practitioners.* By Ansley Wilcox. Reprint from the *Medical Press.*

*Souvenir of the New York Odontological Society.* 1886.

*Annual Address before the Massachusetts Dental Society.* By D. B. Ingalls, D. D. S.

*Homœopathy as Viewed by a Member of the Massachusetts Medical Society.* By Vincent G. Bowditch, A. B., M. D. Reprint from the *Boston Med. and Surg. Journal.*

*Enucleation with Transplantation and Reimplantation of Eyes.* By Charles H. May, M. D. Reprint from the *Medical Record.*

